# Differences in Weight, Hierarchy, and Incidence of Lameness between Two Groups of Adult Pigs Derived from Assisted Reproductive Technologies

**DOI:** 10.3390/ani12243578

**Published:** 2022-12-17

**Authors:** Jon Romero-Aguirregomezcorta, Lisette L. Ramírez, Alba Ortín, Guillermo Ramis, Raquel Romar, Pilar Coy

**Affiliations:** 1 Physiology of Reproduction Group, Department of Physiology, Faculty of Veterinary Medicine, International Excellence Campus for Higher Education and Research (Campus Mare Nostrum), University of Murcia, 30100 Murcia, Spain; 2 Instituto Murciano de Investigación Biosanitaria (IMIB), 30120 Murcia, Spain; 3 Department of Animal Medicine and Surgery, Faculty of Veterinary Medicine, International Excellence Campus for Higher Education and Research (Campus Mare Nostrum), University of Murcia, 30100 Murcia, Spain; 4 Department of Animal Production, Faculty of Veterinary Medicine, International Excellence Campus for Higher Education and Research (Campus Mare Nostrum), University of Murcia, 30100 Murcia, Spain

**Keywords:** lameness, pig, assisted reproduction, adenosine deaminase, growth, phenotype

## Abstract

**Simple Summary:**

More than 90% of the animals in current pig farms have been conceived by artificial insemination (AI). However, it is expected that the number of pigs derived from in vitro fertilization (IVF) will increase in the coming decades since the trade of cryopreserved pig embryos can be a reality soon, as it already is in cattle. Today, nothing is known about the repercussions that IVF could have on animal health, welfare, or food safety because no colonies of study exist yet. We created a small one 4 years ago and the present work shows some data collected from 16 pigs derived from IVF and 14 pigs derived from AI, conceived from the same boar, and housed and fed under the same conditions since they were born. The results point out that the IVF animals showed a lower incidence of lameness and higher weight and occupied more predominant positions in the hierarchy of the herd than the AI animals. Although genetic differences from the maternal line could explain some of these results, it is also possible that the way in which the animals were conceived (AI or IVF) induced better adaptative abilities in the IVF group, explaining the lower incidence of lameness. Nonetheless, more studies with a higher number of animals are necessary to reach consistent conclusions about the phenotype of IVF-derived adult pigs.

**Abstract:**

The in vitro production (IVP) and subsequent transfer of embryos (ET) to recipient mothers is not yet an established reproductive technology in the pig industry, as it is in cattle. However, that the trade of IVP-cryopreserved pig embryos is expected to start in the next decades. Society and governments are increasingly aware of the repercussions that IVP could have for animal health, welfare, behavior, or food safety, but proven scientific information for this type of animal does not exist, since no colonies of pigs have been created to this end. We created a small one and studied the differences between 16 IVP-derived pigs and 14 pigs derived from artificial insemination (AI), at 3.5 years of age, conceived from the same boar, and housed and fed under the same conditions since they were born. Incidence of lameness, position in the herd hierarchy, weight, adenosine deaminase activity, and hematological and biochemical analytes were compared between the two groups of animals. The results showed that the IVP animals weighed more, occupied higher positions in the herd hierarchy, and had a lower incidence of lameness. Although genetic differences from the maternal line could explain some of these results, it is also possible that the IVP animals developed better adaptative abilities, but more studies with a higher number of animals are necessary to reach consistent conclusions.

## 1. Introduction

Although more than 90% of sows have become successfully pregnant by artificial insemination (AI) in modern farms for at least three decades [1], the in vitro production (IVP) and subsequent transfer of embryos (ET) are far less developed technologies in this species. However, advances in their efficiency are continuous, and it is expected that the trade of pig embryos will become a reality in the next decades [2], and that herds of IVP-derived pigs will become part of commercial farms. In this context, it becomes necessary to generate new knowledge about the expected health and yield of these animals, particularly at adult age, since adult pigs derived from the IVP procedures are inexistent today. To this end, we generated a colony of IVP- and AI-derived pigs from the same paternal line and let them grow until natural death with the objective of comparing two of the phenotypical traits of major interest interest for the industry, namely, weight and incidence of lameness; one behavioural parameter to assess their rank in the herd hierarchy; different hematological and biochemical analytes, as markers of health status; and the activity in saliva of adenosine deaminase, a proposed biological marker related to stress. 

Incidence of lameness was selected as a parameter of study because it is the second most important cause of losses in the pig industry after reproductive problems, representing a 15% loss in mothers and finishers [3]. It affects animal welfare and results in economic losses to pork producers. It is expected that lameness will become increasingly relevant as farms are adapted to animal welfare requirements, such as the group housing of pregnant sows and gilts.

A second parameter of productive interest is weight. Previous studies have shown that high weight or fast growth positively correlates with leg weakness and osteochondrosis [4,5]. More specifically, it was noted that pigs with clinical signs of leg weakness grew faster in the early stages of life than pigs without these signs [6]. However, under commercial conditions, the time of slaughter is around 2.5–3 years for the breeding boars and 3.5–4 years for the mothers and, by this time, the pigs have slowed down their growth or balanced their weight by reducing food intake due to the stress caused by the lameness. This makes it very difficult to predict if lameness in adulthood would depend on growth rates in youth. Leg disorders, on the other hand, have a critical influence on the longevity of breeders. In fact, a genetic correlation of 0.32 between the overall leg action and the length of productive life was reported in some swine populations [7]. The animals at the created colony were tracked from birth, and parameters such as weight and average daily gain (ADG) were periodically registered, making them a good model to find out if a correlation with lameness exists.

The behavioural parameter chosen for to be assesed was the ranking of each animal in the feeding line. The current feeding machines in the sows’ stables or farmyards, with free access to food 23 h and 45 min a day (15 min are daily necessities for the system resetting functions), represent a useful tool to study this parameter. The machines register the amount of food consumed and the animal weight every time they enter in, as well as the order of the line they spontaneously establish when deciding to eat. This order represents an objective way to measure the dominant or submissive position that each sow occupies in the herd, and downturns in that position are associated with discomfort, heat, stress, or sickness. Therefore, the study colony would also be useful to find out if IVP-ET animals show a more or less dominant position in the hierarchy than AI animals.

Biochemical and hematological analytes provide very useful information about the health status of the animals and were chosen in the present study to contribute the characterization of the IVP and AI phenotypes of adult pigs. Finally, an attempt to find a good stress biomarker was made. There are certain management practices or inappropriate housing conditions that cause pain and stress to pigs, affecting their performance [8]. In terms of production, stress harms animal welfare and health, resulting in economic losses due to increased mortality, reduced animal growth, reduced reproductive efficiency, and poorer meat quality [9]. The presence of lesions such as gastroesophageal ulceration, lung lesions or foot lesions in the slaughterhouse are common signs of stress, but this assessment is postmortem and does not provide tools that can immediately help the animals in a group [10]. In order to make such an assessment more objective, it is necessary to monitor the biological markers that are related to stress, such as glucocorticoids or adenosine deaminase (ADA), among others [11]. Nowadays, most tests require blood to be taken, but the handling of animals to this end causes acute stress, which is particularly negative and frequent in swine [12]. The use of saliva as a biological sample is a good alternative to blood collection because it is a non-invasive method. ADA is an enzyme considered a biomarker of the immune system, found in serum and saliva, as well as in lymphoid tissue, where it is necessary for monocyte-to-macrophage differentiation and for the development of B- and T-lymphocytes [13]. The role of this enzyme in the immune system is proven by the fact that its deficiency produces immune dysfunction [14]. It has been suggested that there is a direct relationship between the ADA levels in saliva and the immune status of an animal [15], but, for the moment, there is little information on the possibility of using ADA as a biomarker of other parameters of interest for animal production, such as weight, the order of hierarchy in the herd, or the incidence of locomotor problems. 

For all the above-mentioned reasons, we hypothesized that the IVP and AI animals at 3.5 years of age could show differences in their incidence of lameness, weight, hierarchy order, hematological and biochemical parameters, or the ADA activity in their saliva that could help to phenotype ART-derived pigs in adulthood.

## 2. Materials and Methods

### 2.1. Ethics

The experimental work performed in this study was submitted for evaluation by the Animal Experimentation Ethics Committee (CEEA) of the University of Murcia. After approval, authorization to carry out experiments with animals was granted by Dirección General de Agricultura, Ganadería, Pesca y Acuicultura, Región de Murcia (A13170706).

### 2.2. Animals

Animals were handle following the current regulations on animal protection and pig farm management. The general health status of the animals was supervised daily by an experienced technician.

The population under study consisted of thirty 3.5-year-old pigs, 25 females and 5 males, housed in the sanctuary of the Veterinary Teaching Farm, Veterinary Faculty, University of Murcia, Murcia (Spain). These animals were obtained as a result of a previous study which aimed to compare the impact of using in vitro production and embryo transfer (IVP-ET) or AI on the phenotype of the offspring during its first days of age, in terms of their growth and blood parameters [16]. AI-derived animals were purebred Large White (LW) individuals, while the IVP animals were the result of crossing purebred LW semen of the same boar as for the AI animals with oocytes from finishing gilts, resulting from mating Duroc or Pietrain boars with LW × Landrace sows. Until all of the animals reached two years of age, males and females were housed and fed under the same conditions and water was provided ad libitum. At the age of two, females were trained to use a feeding machine (Compident, Schauer-Agrotonic, Prambachkirchen, Austria), which allowed free-range sows to feed on demand. The system was equipped with a weigh scale for individual feeding according to the animal condition and was computer-controlled via TOPO software (Schauer-Agrotonic, Prambachkirchen, Austria). The daily intake, number of entries, order of entry, and individual weight of each animal were recorded by the system. 

### 2.3. Data Collection

The weight of the animals was collected from birth until day 45 with a hanging weight scale; from day 84 forward it was recorded with a Baxtran BR 15 weight scale (Giropès SL, Vilamalla, Spain). From the age of two, after some training to learn how to enter into the feeding machine, females were weighed automatically, and the data were collected and stored by the TOPO software. Average daily gain (ADG) was calculated by subtracting the body weight of the previous assessment and dividing by the days passed between dates. Hierarchy was assessed as the order in which each sow entered the feeding machine and ranged from 1st to the 25th position. The TOPO system provided these data. The sows usually entered the feeding machine at night, just a few minutes after the resetting of the machine that takes place from 00:00 to 00:15 h. No feeding machine was employed for the boars, as males exceeded the entry size limitations. This is the reason why no information on the individual weight, ADG, or hierarchy is available for males. 

### 2.4. Blood Sampling, Hematological and Biochemical Data

The blood samples at 3.5 years of age were collected by direct venipuncture of the external jugular vein with a 18 G × 25 mm needle and lithium heparin tubes (BD Vacutainer, BD, Madrid Spain). The blood tubes were transported to the laboratory, and hematological analysis was performed using a hematology analyzer (Advia 120, Siemens Healthcare GmbH, Erlangen, Germany). The parameters analyzed were hematocrit (HCT, %), hemoglobin (HB, g/dL), concentration of red blood cells (RBC, ×10^9^/mL), concentration of white blood cells (WBC, ×10^6^/mL), neutrophils (×10^6^/mL), and lymphocytes (×10^6^/mL). Then, the blood was centrifuged at 1008× *g* for 10 min at room temperature, and biochemical analysis was performed using a chemistry analyzer for plasma (Olympus AU400, Tokio, Japan). The biochemical parameters analyzed were total protein (TP, g/dL), albumin, (ALB, g/dL), globulin (GLO, g/dL) and glucose (GLU, mg/dL).

### 2.5. Determination of Adenosine Deaminase (ADA) in Saliva

Saliva was collected from the animals using Salivette cotton swabs (Sarstedt, Nümbrecht, Germany). The procedure was as follows: the sponge was placed on a thin, flexible metal rod and introduced into the pig’s mouth, where the pig was allowed to chew it until the sponge was completely moistened, and subsequently introduced into the Salivette tube. Once the samples were obtained, the saliva tubes were placed in an isothermal box until their arrival at the laboratory, where they were centrifuged at 3500× *g* for 10 min at 4 °C. The supernatant was collected in Eppendorf tubes of 1.5 mL, and the sediment discarded. The saliva specimens were analysed right after centrifugation, while duplicates were stored at −80 °C in case a second analysis was required.

The ADA activity determination was performed using a commercial ADA assay kit (DZ117A, Diazyme Laboratories Inc., Poway, CA, USA). Briefly, the method is based on the enzymatic deamination of adenosine to inosine, which is converted to hypoxanthine by purine nucleoside phosphorylase. Hypoxanthine is then converted to uric acid and hydrogen peroxide (H_2_O_2_) by xanthine oxidase. H_2_O_2_ is further reacted with N-Ethyl-N-(2-hydroxy-3-sulfopropyl)-3-methylaniline and 4-aminoantipyrine in the presence of peroxidase to generate quinone dye, which is monitored in a kinetic manner and controlled by a wavelength of 550 nm. The method, previously validated for pig saliva [13], was adapted to the automatic analyzer Olympus AU600 (Beckman Coulter Inc, Brea, CA, USA) following the manufacturer’s protocol with some modifications. Because undiluted saliva samples yielded results out of the dynamic range of the method, a 1:8 dilution (1 volume of saliva sample and 7 volumes of distilled water) was applied in all samples and the results were multiplied by 8.

### 2.6. Lameness Diagnosis

An experienced veterinarian performed a weekly visual observation of the animals to identify lameness. The following features were taken into account for the diagnosis [17]: behavioral aspects (presence of lameness, head nod, weight bearing on the affected limb, or the ability or willingness to ambulate) and physical aspects (presence of injuries or swelling in hooves, joints or limbs, presence of abscesses or radiographic alterations). No cases of osteochondrosis were diagnosed among the lame animals; by contrast, when the content of the abscesses was aspirated and analyzed, the presence of Aerococcus viridans was identified in all of the cases.

### 2.7. Statistical Analysis

The statistical analysis of the data was performed with R software (version 4.1.2, R Core Team 2021, Vienna, Austria). The incidence of lameness in both the AI and the IVP group was analyzed by Chi-Square, and the value for Fisher’s exact test was used to denote the dependency between variables. The differences in weight and in hematological and biochemical parameters between groups, and between healthy and lame sows, were analyzed by ANOVA since the assumptions of homogeneity of variances and normality were met. Non-parametric bilateral correlations (Spearman’s Rho) were used to study the differences between the remaining pairwise combinations of the variables (group, weight, incidence of lameness, ADG at different time intervals, hematological and biochemical parameters, and ADA). The correlation coefficients (s) were interpreted as follows: s < |0.1|, negligible effect; |0.1| < s < |0.3|, small effect; |0.3| < s < |0.5|, medium effect; and s > |0.5|, large effect.

## 3. Results

### 3.1. Incidence of Lameness in AI-Derived and IVP- Derived Pigs of the Colony

When the incidence of lameness in the colony of animals (N = 30) was analyzed, the percentage of affected AI-derived animals was 85.71% (12/14) vs. 31.25% (5/16) in the IVP group. With these data, Fisher’s exact test (0.004) denoted that this variable depended on the group and that the animals derived from AI had a higher incidence of lameness at 3.5 years of age than the animals from IVP. This first finding was unexpected due to the genetic similarity among the groups and it was decided to deepen the search for possible causes of such a difference, starting with weight at present and growth rate during the first year of life.

### 3.2. Differences in Weight between Groups (AI- and IVP- Derived Sows) and between Health Status (Healthy and Lame Sows); Correlations between Lameness and Growth Rate

**Figure 1:**
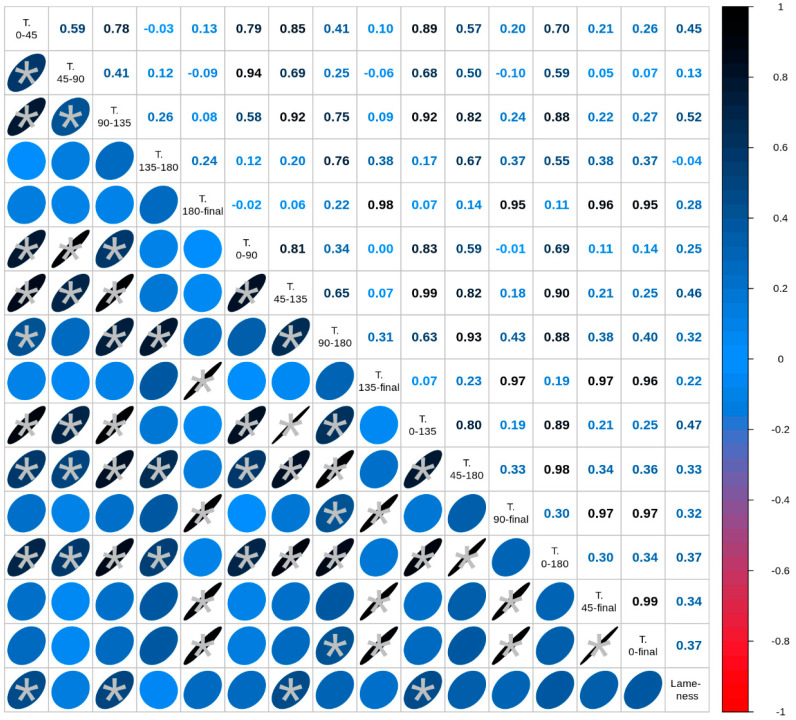
Correlations between average daily gain, calculated at different time intervals (T), and lameness at 3.5 years of age in a colony of sows (N = 25) produced by assisted reproductive technologies. The matrix shows the Spearman’s Rho correlation. The color and form of the ellipse indicate the correlation strength (s). Red means negative and blue means positive correlation. The rounder the ellipse the lower the correlation. The asterisk indicates a significant correlation between the two parameters (*p* < 0.05).

The weight of the animals from AI was significantly lower (*p* < 0.001) than that of the IVP animals at 3.5 years of age (Table 1). Also, significant differences (*p* < 0.001) were found in the weights of healthy and lame sows (Table 2), confirming the previous observation that AI-derived animals, despite being those with the lowest weights, had the highest incidence of lameness (weight at birth and during the postnatal growth provided in Supplementary Table S1). Moreover, the growth rate (calculated as the average daily gain [ADG] at different time intervals during the first year of life) had a large effect on the incidence of lameness (*s >* |0.5|) for the growth interval of 90–135 days and a medium effect (|0.3| *< s <* |0.5|) for the growth intervals of 0–45, 45–135, 90–180, 0–135, 45–180, 90–365, 0–180, 45–365, and 0–365 days (Figure 1). These results suggest that, although fast growth is associated with the incidence of lameness [18], and both AI and IVP-derived animals grew fast and at similar rates (see data from Paris-Oller [16,19]), the IVP group was in some way more resistant to suffering from lameness in adulthood.

### 3.3. Differences in the Hierarchy between AI- and IVP-Derived Animals. Correlation with Lameness

Hierarchy in the herd, assessed as the order occupied by each sow in the feeding line, was different between groups (s = −0.481, *p* < 0.05), correlating inversely with weight (s = −0.596, *p* < 0.01) and directly (s = 0.566, *p* < 0.01) with lameness. That means that the AI animals, showing lower weight and higher incidence of lameness, occupied the lowest positions in the hierarchy. 

### 3.4. Differences in Adenosine Deaminase Activity (ADA) between AI- and IVP- Derived Animals and Correlation with Lameness, Hierarchy, and Weight

The activity of the enzyme ADA, assessed in saliva, was not different between the AI- and IVP-derived pigs (Table 3 and Table S2). Similarly, ADA did not correlate either with the weight, hierarchy, or health status of the animals (Supplementary Table S3).

### 3.5. Differences in Biochemical and Hematological Data from Pigs Derived from AI or IVP at 3.5 Years of Age and Correlation with ADA Activity

Regarding biochemical data, TP, ALB, GLO, and GLU values (Table 4) fell within the physiological range for healthy pigs [19,20], and no significant differences were found for these parameters between AI- and IVP-derived animals.

On the hematological data, the HCT, HB, RBC, WBC, NEU, and LYM values (Table 5) fell within the physiological range for healthy animals [21,22]. No significant differences were found for HCT, HB, RBC, or LYM between AI- and IVP-derived animals. However, AI-derived animals showed a significantly higher WBC (*p* = 0.015) and NEU (*p* = 0.010) than their IVP counterparts.

Similarly to weight, hierarchy, and lameness, ADA did not correlate with any of the biochemical or hematological parameters (Supplementary Tables S4 and S5, respectively).

## 4. Discussion

The data in this study are extremely valuable because no other colony of IVP- and AI-derived pigs from the same paternal line exists, to our knowledge, in the world. The differences observed, even with only 30 animals, were highly significant for some of the parameters under study and gave relevant information about the phenotype of adult animals derived from ART. Nonetheless, wider studies with a larger number of animals are necessary in the future to corroborate the present findings.

In order to interpret the data, we have to keep in mind that we compared the results for pure animals versus animals with at least a two-way cross. More than 40 years ago, it was found that 3-way crosses with LW grow significantly faster and have more efficient feed conversion rates than purebred animals or animals obtained by back-cross [23,24]. In fact, our data showed a difference of 17% in the average weight of the AI animals compared to the IVP animals, which could be attributed to maternal heterosis [25]. However, we cannot rule out the possibility that the effect of crossbreeding may be compounded by the effect of epigenetic marks resulting from the in vitro fertilization and culture of the embryos [26], and that a cumulative effect may have been produced, affecting the differences in growth between groups.

As in the case of growth, the observation that lameness was more frequent in the AI animals than in the IVP animals made us hypothesize that genetics was the reason. Given that (i) among the different WBC, NEU lead the first wave of host defence against infection, acting as powerful effector cells that destroy infectious threats through phagocytosis, degranulation, reactive oxygen species and neutrophil extracellular traps [27]; (ii) we found a significant increase in the concentration of NEU and WBC in the AI animals; and (iii) the animals had, in most cases, suffered from infectious osteomyelitis or osteoarthritis, we could speculate that the greater hybrid vigor of the IVF animals has been an adaptive advantage, conferring greater disease resistance. 

According to the Developmental Origin of Health and Disease (DOHaD) hypothesis [28], the differences in the environment during the preimplantation period in the two groups of animals can draw marks in the epigenome of the embryos, such as alterations in the DNA methylation patterns [29],whose consequences are expressed later in development. It is well known that the plasticity embryos lets them adapt to stressful conditions such as those derived from the in vitro culture, and it has been shown in mice that the long-term programming of postnatal development, growth, physiology, and behavior is affected irreversibly by preimplantation conditions [30,31]. Also, studies in pig blastocysts have shown differences in the DNA methylation and gene expression profiles of embryos grown in different culture systems [26]. We hypothesize that the epigenetic programming of the IVP-derived animals from the present study during the in vitro embryo culture let them adapt better to the hostile environmental conditions and could contribute to their fast growth and lower incidence of lameness. 

Not only this, but the IVP-derived animals also showed a higher weight at birth, during the first year of life [19], and at 3.5 years of age (present data). Previous data have shown that pigs with leg problems were usually heavier and had more back fat compared to healthy pigs [18]. Again, our results disagree with this information and lead us to the option that our IVP-derived animals were in some way protected or better adapted since their early days than the AI-derived ones. The difference in weight could also result from a reduced food intake among lame animals due to their disease. Lameness often leads to a marked loss of welfare, hinders the animals’ access to feed, and takes away their advantages in maintaining hierarchies or fighting for resources. This is reinforced by the finding that animals with lameness occupy a low position in the hierarchical scale.

Indeed, an additional proof for such a better adaptation was found in the behavioral parameter we used to assess the rank in the herd hierarchy: again, the positions occupied by the IVP animals in the feeding line were usually higher than those of the AI animals since, despite the fact that the machine was open and available for 23 h and 45 min per day, all of the animals decided to eat at the same time every day.

Altogether, the results indicate that the AI-derived animals at 3.5 years of age weighed less, showed a higher incidence of lameness, and occupied lower positions in the hierarchy of the herd than the IVP animals under the same conditions. These findings were unexpected since the whole group was treated exactly the same since they were born. In an attempt to find an innocuous biomarker for health status (lameness or not) or stress (caused by pain due to lameness), we decided to measure ADA in saliva. Kaiser et al. (2021) found an increase in ADA in lame pigs [32], and another research study showed that this enzyme is increased in the case of inflammatory diseases [33]. Considering that inflammation can be a cause of lameness, it is understandable that ADA activity would increase when such pathology is present. Furthermore, considering the enzyme as a biomarker of stress, it should show some correlation with other variables studied, since animals suffering from lameness not only show pain but also suffer from stress and discomfort, causing the biomarkers related to these symptoms to increase [34].

In our colony, ADA did not show any correlation with lameness, neither with weight nor hierarchy. It is possible that this difference between our results and those of other authors is due to the chronicity of lameness in our animals. On most farms, when lameness is treated once and no recovery is observed, the animals are slaughtered. In our case, due to the type of colony and the high value of the individuals, the treatment of lameness was much more thorough, including specific antibiograms and anti-inflammatory treatments (i.e., intramuscular injection of 10 mg de spectinomycin and 5 mg de lincomycin per kg of body weight per day for 3–7 days, and 3 mg ketoprofen per kg of body weight in a single dose), feed and water medication, hoof trimming, and floor hygiene, among other measures, which allowed most of the animals to recover from the acute process and pain, and go on to live normal lives even though the lameness remained chronic. As indicated by Contreras-Aguilar et al., 2019 [34], the concentration of ADA correlates with animals with pain and lameness, but not with animals with lameness that do not show pain, as it is in the current case. 

## 5. Conclusions

The results of the present study show phenotypical differences between two groups of adult pigs identically managed but produced by two different assisted reproductive technologies, AI and IVP-ET. Since, while more than 90% of the pigs in modern farms derive from AI, an increase in the number of IVP animals is expected in the near future, these results acquire particular relevance. Although limited in number, this is the first animal colony of adult pigs produced by IVP tracked since birth, and the evidence here, showing that they have a lower incidence of lameness, higher weight, and higher position in the herd hierarchy than AI-derived animals, could come to be of special significance in the design of future farm programs. 

## Figures and Tables

**Table 1:** Weight at 3.5 years of age in sows derived from artificial insemination (AI) and in vitro embryo production (IVP). Data are expressed as mean ± s.e.m.

	AI	IVP
N	11	14
Weight (kg)	202.10 ± 4.27 ^a^	245.64 ± 6.03 ^b^

^a,b^ denote significant differences (*p* < 0.001).

**Table 2:** Weight at 3.5 years of age of healthy and lamesows. Data are expressed as mean ± s.e.m.

	Lame	Healthy
N	13	12
Weight (kg)	209.92 ± 5.03 ^a^	244.42 ± 8.24 ^b^

^a,b^ denote significant differences (*p* < 0.001).

**Table 3:** Adenosine deaminase activity (ADA) at 3.5 years of age in pigs derived from artificial insemination (AI) and in vitro embryo production (IVP). Data are expressed as mean ± s.e.m.

	AI	IVP
N	14	16
ADA (IU/L)	1552 ± 5922	2027 ± 813

**Table 4:** Biochemical data from pigs derived from artificial insemination (AI) and in vitro embryo production (IVP) at 3.5 years of age. The parameters analyzed were total protein (TP), albumin (ALB), globulin (GLO), and glucose (GLU). Data are expressed as mean ± s.e.m.

Variable	AI	IVP
N	14	16
TP (g/dL)	7.88 ± 0.19	7.98 ± 0.14
ALB (g/dL)	3.46 ± 0.12	3.64 ± 0.09
GLO (g/dL)	4.42 ± 0.20	4.34 ± 0.17
GLU (mg/dL)	60.47 ± 2.11	61.61 ± 2.58

**Table 5:** Hematological data from pigs derived from artificial insemination (AI) and in vitro embryo production (IVP) at 3.5 years of age. The parameters analyzed were hematocrit (HCT), hemoglobin (HB), concentration of red blood cells (RBC), concentration of white blood cells (WBC), neutrophils (NEU), and lymphocytes (LYM). Data are expressed as mean ± s.e.m.

Variable	AI	IVP
N	14	16
HCT (%)	39.09 ± 1.18	37.82 ± 0.98
HB (g/dL)	13.48 ± 0.42	13.01 ± 0.35
RBC (×10^9^/mL)	6.29 ± 0.19	6.04 ± 0.15
WBC (×10^6^/mL) *	13.25 ± 0.88	10.89 ± 0.38
NEU (×10^6^/mL) **	7.46 ± 0.66	5.46 ± 0.36
LYM (×10^6^/mL)	4.26 ± 0.23	4.11 ± 0.14

* *p* = 0.015; ** *p* = 0.010.

## Data Availability

The data presented in this study are openly available at the University of Murcia-Physiology of Reproduction research group website (www.um.es/fisiorep/index.html (accessed on 14 December 2022)).

## Supplementary Materials

The following supporting information can be downloaded at: https://www.mdpi.com/article/10.3390/ani12243578/s1. Table S1: Weight at birth and during the postnatal growth in sows derived from artificial insemination and in vitro embryo production. Table S2: Individual data on the adenosine deaminase activity at 3.5 years of age in pigs derived from artificial insemination and in vitro embryo production. Table S3: Spearman’s rho correlation and significance between adenosine deaminase activity and weight of animals, hierarchy, and health status in sows derived from artificial insemination and in vitro embryo production at 3.5 years of age. Table S4: Spearman’s rho correlation and significance between adenosine deaminase activity and biochemical parameters: total protein, albumin, globulin, and glucose; from pigs derived from artificial insemination and in vitro embryo production at 3.5 years of age. Table S5: Spearman’s rho correlation and significance between adenosine deaminase activity and hematological parameters: hematocrit, hemoglobin, concentration of red blood cells, concentration of white blood cells, neutrophils, and lymphocytes; from pigs derived from artificial insemination and in vitro embryo production at 3.5 years of age.
