# Supplementary material for: Differences in Weight, Hierarchy, and Incidence of Lameness between Two Groups of Adult Pigs Derived from Assisted Reproductive Technologies
Source: Animals (Basel). 2022 Dec 17;12(24):3578. doi: 10.3390/ani12243578 (PMC9774099; doi:10.3390/ani12243578)
Supplement: Supplementary file 1 [file animals-12-03578-s001.zip › animals-2087015-supplementary.pdf]

Supplementary Table S1. Weight at birth (D0) and during the postnatal growth (Days 3 to 180; D3-180) in sows derived from artificial insemination (AI) and *in vitro* embryo production (IVP). Data are expressed as mean ± s.e.m.

		AI	IVP
**N**		**11**	**14**
**Weight (kg)**	D0	1.08 ± 0.07[a]	1.38 ± 0.09[b]
	D3	1.22 ± 0.07[a]	1.96 ± 0.13[b]
	D9	2.02 ± 0.17[a]	3.38 ± 0.20[b]
	D15	3.03 ± 0.19[a]	4.93 ± 0.33[b]
	D30	4.90 ± 0.25[a]	8.27 ± 0.52[b]
	D45	7.45 ± 0.30[a]	11.52 ± 0.72[b]
	D60	12.18 ± 0.64[a]	19.73 ± 1.06[b]
	D75	19.60 ± 0.73[a]	30.79 ± 2.02[b]
	D90	29.89 ± 1.24[a]	41.41 ± 2.23[b]
	D105	40.00 ± 1.57[a]	55.61 ± 3.12[b]
	D120	54.50 ± 1.99[a]	69.96 ± 3.45[b]
	D135	65.55 ± 2.23[a]	85.04 ± 3.62[b]
	D150	79.45 ± 2.21[a]	98.89 ± 3.61[b]
	D165	91.23 ± 2.32[a]	115.18 ± 4.96[b]
	D180	102.95 ± 2.37[a]	124.17 ± 4.52[b]

[a,b] denote significant differences ($P<0.001$).

Supplementary Table S2. Individual data on the adenosine deaminase activity (ADA) at 3.5 years of age in pigs derived from artificial insemination (AI) and *in vitro* embryo production (IVP).

ANIMAL ID (FEMALES)	ADA (IU/L)	ANIMAL ID (MALES)	ADA (IU/L)
AI 505	8920	AI 538	356.9
AI 513	1218.4	AI 539	165.8
AI 520	2502.4	AI 553	304.2
AI530	1251.8	IVP 302	689.2
AI532	1643	IVP 316	238.2
AI534	570.6		
AI541	579.4		
AI545	1331.2		
AI551	1449.8		
AI557	662.4		
AI548	767.8		
IVP 100	1219.2		
IVP 101	642.6		
IVP 102	13622.4		
IVP 103	841		
IVP 110	1069.8		
IVP 111	422.2		
IVP 114	1977.2		
IVP 121	522.2		
IVP 122	3857.6		
IVP 124	1382		
IVP 303	614.6		
IVP 307	3126		
IVP 313	1688		
IVP 318	523.4		

Supplementary Table S3. Spearman's rho correlation (s) and significance (*P*) between adenosine deaminase activity (ADA) and weight of animals, hierarchy, and health status in sows derived from artificial insemination (AI) and *in vitro* embryo production (IVP) at 3.5 years of age.

		**Weight**	**Hierarchy**	**Health status**
	**N**	25	25	25
**ADA**	**s**	-0.152	-0.143	0.322
	P	0.467	0.495	0.117

Supplementary Table S4. Spearman's rho correlation (s) and significance (*P*) between adenosine deaminase activity (ADA) and biochemical parameters: total protein (TP), albumin (ALB), globulin (GLO) and glucose (GLU), from pigs derived from artificial insemination (AI) and *in vitro* embryo production (IVP) at 3.5 years of age.

		**TP**	**ALB**	**GLO**	**GLU**
	**N**	30	30	30	30
**ADA**	**s**	- 0.065	- 0.352	0.223	0.049
	P	0.732	0.057	0.237	0.798

Supplementary Table S5. Spearman's rho correlation (s) and significance (P) between adenosine deaminase activity (ADA) and hematological parameters: hematocrit (HCT), hemoglobin (HB), concentration of red blood cells (RBC), concentration of white blood cells (WBC), neutrophils (NEU), and lymphocytes (LYM); from pigs derived from artificial insemination (AI) and *in vitro* embryo production (IVP) at 3.5 years of age.

		**HCT**	**HB**	**RBC**	**WBC**	**NEU**	**LYM**
	**N**	30	30	30	30	30	30
**ADA**	**s**	- 0.238	- 0.222	- 0.241	0.047	0.028	0.095
	P	0.206	0.239	0.199	0.805	0.882	0.619
